# Nanda-Hamner Curves Show Huge Latitudinal Variation but No Circadian Components in *Drosophila Montana* Photoperiodism

**DOI:** 10.1177/0748730421997265

**Published:** 2021-03-22

**Authors:** Pekka Lankinen, Chedly Kastally, Anneli Hoikkala

**Affiliations:** * Department of Ecology and Genetics, University of Oulu, Oulu, Finland; † Department of Biological and Environmental Science, University of Jyväskylä, Jyväskylä, Finland

**Keywords:** reproductive diapause, photoperiodic counter, circadian clock, non-circadian photoperiods, northern insects

## Abstract

Insect species with a wide distribution offer a great opportunity to trace latitudinal variation in the photoperiodic regulation of traits important in reproduction and stress tolerances. We measured this variation in the photoperiodic time-measuring system underlying reproductive diapause in *Drosophila montana*, using a Nanda-Hamner (NH) protocol. None of the study strains showed diel rhythmicity in female diapause proportions under a constant day length (12 h) and varying night lengths in photoperiods ranging from 16 to 84 h at 16°C. In the northernmost strains (above 55°N), nearly all females entered diapause under all photoperiods and about half of them even in continuous darkness, while the females of the southern strains showed high diapause proportions only in the circadian 24 h photoperiod. Significant correlation between the strains’ mean diapause proportions in ≥ 24 h photoperiods and critical day length (CDL; half of the females enter diapause) suggests at least partial causal connection between the traits. Interestingly, females of the northern strains entered diapause even in ≤ 24 h photoperiods, where the night length was shorter than their critical night length (24 h - CDL), but where the females experienced a higher number of Light:Dark cycles than in 24 h photoperiods. NH experiments, performed on the control and selection lines in our previous selection experiment, and completed here, gave similar results and confirmed that selection for shorter, southern-type CDL decreases female diapausing rate in non-circadian photoperiods. Overall, our study shows that *D. montana* females measure night length quantitatively, that the photoperiodic counter may play a prominent but slightly different role in extra short and extra long photoperiods and that northern strains show high stability against perturbations in the photoperiod length and in the presence of LD cycles. These features are best explained by the quantitative versions of the damped external coincidence model.

Correct timing of development and progeny production is of special importance for species living in a seasonally varying environment. Many insects from high latitudes enter diapause at the beginning of winter at a species-specific life stage, either as egg, larva, pupa or adult, which increases their possibilities to survive over this season and/or to postpone their progeny production to a more favorable season (Hahn and Denlinger, 2007). In photoperiodic reproductive diapause, female ovaries remain undeveloped if the day length before or during their emergence is shorter than the critical day length (CDL) for diapause induction (a photoperiod where half of the females of a given population enters diapause).

Insects’ daily and seasonal rhythms rest on the function of two timers, the circadian clock system and the photoperiodic time measurement, PPTM. Circadian clock(s) maintain rhythmicity in a wide range of behavioral, physiological, and metabolic processes, while PPTM plays a central role in regulating photoperiodically controlled seasonal activities, like diapause (Vaz Nunes and Saunders, 1999; Koštál, 2011; Denlinger et al., 2017). PPTM consists of light receptors, photoperiodic clock, and counter and endocrine effectors leading to diapause or nondiapause developmental pathways (Saunders, 2010). In this timer, the clock measures the night length quantitatively or determines whether it is longer or shorter than the critical night length (CNL, 24 h - CDL), while the counter accumulates information on the number of successive long (or short) nights (Saunders, 1981, 2002). In the hourglass model, lacking any circadian components, night length measurement is based on the accumulation of a hypothetical chemical substance (“diapause titer”) during the dark period (Lees, 1973). In the internal and external coincidence models, PPTM is expected to involve non-damping or damping circadian oscillator(s) operating either at night length measurement or at the counter level. In the internal coincidence model, PPTM is based on seasonal changes in the phase relationship between two circadian oscillators, the morning (M) and the evening (E) oscillator, while in the external one the night length is defined as long or short depending on whether the photoinducible phase (φ), oscillating in 24 h cycle, coincides regularly with a light or a dark period (Pittendrigh, 1972; Vaz Nunes and Veerman, 1982; Vaz Nunes and Saunders, 1999). However, in the quantitative versions of external model the evaluation of long night cycles rests on the accumulation of a diapause titer, as in the hourglass model. The amount of this titer is expected to increase during the dark periods and decrease during the light periods, so that the diapause induction occurs when the substance reaches an internal reference threshold (Saunders, 1981; Meuti and Denlinger, 2013).

Nanda-Hamner (NH) experiments can be used to detect the presence of circadian components in the PPTM of the study species (Vaz Nunes and Saunders, 1999). In this method, the proportion of insects giving a short-day response is typically measured in a set of experiments with the day length of 12 h and the night length varying from 4 to 72 h (Nanda and Hamner, 1958). In internal and external coincidence models, insects’ sensitivity to light is expected to vary between low (insensitivity) and high levels in cycles of about 24 h, so that the coincidence of their photoinducible phase(s) with either darkness or light determines whether they recognize the nights as long or short, respectively (Figure 1). Consequently, regularly cycling peaks in insect diapause incidence under extra long nights are regarded as a sign of circadian oscillation in PPTM (Saunders, 2002). The diel rhythmicity in insect diapause peaks may also reflect the clock-controlled synthesis of diapause inducing substrate (e.g., transcription factors), while the lack of rhythmicity suggests that the substrate may be synthesized constitutively during darkness (Veerman, 2001). NH experiments have revealed 24 h periodicity in the diapause induction of several species, including *Drosophila auraria* (Pittendrigh, 1981) and *Drosophila melanogaster* (Saunders et al., 1989) flies and *Wyeomyia smithii* pitcher plant mosquitoes (Wegis et al., 1997), but failed to do so in species like *Dendrolimus punctatus* pine caterpillars (Huang et al., 2005) and *Drosophila montana* flies (Kauranen et al., 2013). However, the interpretation of NH rhythms may not always be straightforward, as the rhythmic outcome may also be an expression of a circadian disturbance induced by internal desynchronization in unnatural regimes (Veerman, 2001), or it may reflect a part of the circadian system that is independent from night length measurement (Saunders, 2002; Bradshaw et al., 2003).

**Figure 1.**
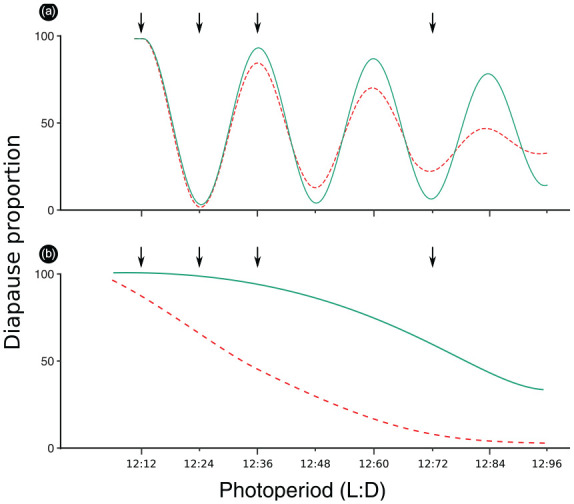
Expected diapause in LD cycles of 12 h light and increasing hours of dark. Arrows indicate the LD cycles used in this study. (a) Positive NH-response: solid and dashed lines represent respectively a slow and a rapid damping of the oscillations. (b) Negative NH-response: solid and dashed lines represent respectively a high and a low incidence of diapause. Abbreviations: LD = Light:Dark; NH = Nanda-Hamner.

Most studies of latitudinal variation in the properties of the circadian oscillators and their involvement in PPTM have been performed on species that display a “positive response” (24 h periodicity; Figure 1a) in NH experiment. For example, Vaz Nunes et al. (1990) found a high correlation between the CNL of *Tetranychus urticae* spider mites and latitude, while the correlation between these traits and the length of the period between mite diapause peaks in NH experiments remained quite low. According to Saunders and Lewis (1987) and Vaz Nunes et al. (1990), these results may be explained by an increase in the “subjective light intensity” (photoreceptor sensitivity) in the northern populations, which could lead to longer CDL without affecting the period of the circadian oscillations underlying PPTM. Moreover, in several species, including *Ostrinia nubilalis* corn borer (Takeda and Skopik, 1985) and *W. smithii* mosquito (Wegis et al., 1997), the amplitude of the rhythmic component of PPTM has been found to decline toward higher latitudes. Bradshaw and Holzapfel (2001) suggest this to result from a decline in the “coupling strength” of the circadian clock system and PPTM, so that an hourglass-type timer may have taken over the function of the circadian-based PPTM in the northern populations. Pittendrigh (1972) also raises a question on whether a high diapause incidence of “northern strains” under prolonged nights could refer to a high stability of female diapause response against perturbations in the photoperiod length.

In *Drosophila montana*, a species of the *virilis* group, the developmental pathway of female ovaries (direct maturation vs diapause) is determined by photoperiodic cues that the females receive after eclosion (Salminen et al., 2012). PPTM of *D. montana* has been suggested either to lack strong oscillators or to be based on heavily damping circadian oscillator(s) of external coincidence model, as the flies of this species have only one activity peak during a day (Kauranen et al., 2012) and as the females do not show circadian periodicity in their diapause proportions in NH experiment (Kauranen et al., 2013). The distribution of *D. montana* populations between the latitudes of 30°N and 70°N on different continents, and the robust latitudinal clines detected in the CDL and its temperature-sensitivity of this species (Tyukmaeva et al., 2020), offer a unique opportunity to trace latitudinal variation in different aspects of PPTM in a species showing a negative NH response. In the present study, we measured latitudinal variation in *D. montana* diapause proportions in circadian (24 h) and non-circadian (extra long and extra short) photoperiods in NH experiment, and compared these proportions to respective variation in the CDLs of the same strains. As study material we used *D. montana* strains originating from different latitudes and the selection and control lines from our earlier selection experiment for shorter CDL (Kauranen et al., 2019). Our main goal was to disentangle the mechanisms driving and maintaining latitudinal variation in the function of PPTM underlying *D. montana* females’ reproductive diapause and to find out whether variation in this trait can be explained by the qualitative or quantitative version of the damped external coincidence model (see Tagaya et al., 2010). The main questions were following. (1) Do female diapause proportions in ≥ 24 h photoperiods show circadian rhythmicity and/or latitudinal variation, and do the females of northern strains show higher stability against perturbations in the photoperiod length than those of the southern strains? (2) Do the strains’ mean diapause proportions in these photoperiods correlate with respective variation in CDL? (3) How do the photoperiodic clock and counter of PPTM function in different photoperiods? Does the clock measure the night length quantitatively or does it only classify it as shorter or longer than the strain CNL, and does the accumulation of daily cycles at counter level lead to a different diapause response depending on the length of the dark period? If *D. montana* diapause induction is based on the qualitative version of the damped external coincidence model, we expect to see a high peak in female diapause proportions in LD (Light:Dark) 12:12 and at least a slight increase in this trait in LDs 12:36. However, if it is based on the quantitative version of this model, we expect to see a diapause peak only in LD 12:12 and to find correlation between CDL and female diapause proportions in NH experiments. In this case, the strains with a long CDL/short CNL should have high diapause in practically all photoperiods, while the ones with a short CDL/long CNL should have a low diapause in all photoperiods except LD 12:12 (see Figure 1b).

## Material and Methods

### Fly Strains

We traced latitudinal variation in *D. montana* females’ CDL and NH responses using 28 strains established from the progenies of single fertilized females, collected from a large scale of latitudes from Europe, North America and Asia (Table 1; Suppl. Fig. S1). We refer to all these strains as cline strains, the strains from above 55°N as northern strains and the ones below 55°N as southern strain (if not defined more precisely). All flies collected in 2002 or later come from our own collection trips and represent an overwintered generation, while information on the collecting time of the founders of the older strains is missing. We included in this study also two strains that had been in laboratory for more than 70 years to see whether they still showed diapause responses typical to their home site. The strains were maintained in continuous light (LL), 16°C ± 1°C and 60%-70% humidity since their arrival to Oulu University, which prevented the females from entering diapause and was optimal for flies’ survival and progeny production. Part of data used for drawing the photoperiod response curves (PPRCs) and estimating the CDLs of 16 of the 28 *D. montana* cline strains has been published earlier (Table 1). Here, we utilized these data, together with data from additional LD cycles for some of the strains, to estimate CDLs for all strains using the same method.

**Table 1.** Collecting site, latitude and collecting year of *D. montana* strains and the codes used for the strains in earlier studies and in the present study.

Collecting Site	Latitude	Collecting Year	Strain Code in Earlier Studies	Strain Code in This Study
Europe				
Oulanka, Finland	66.4	2002	O33^a^	Eu1
Oulanka, Finland	66.4	2008	243OJ8^a^	Eu2
Kemi, Finland	65.7	2002	K15^a,b^	Eu3
Kemi, Finland	65.7	2002	K29^a^	Eu4
Kemi, Finland	65.7	2002	K50^a,b^	Eu5
Pudasjärvi, Finland	65.4	2009	29PJ209^a,b^	Eu6
Paltamo, Finland	64.3	2008	1KJF^a,b,d^	Eu7
Lahti, Finland	61.1	2009	L4, L409^a,b,d,e^	Eu8
Lahti, Finland	61.1	2009	L6^a,b^	Eu9
Lahti, Finland	61.1	2009	L8, L809^a,b,d,e^	Eu10
Lahti, Finland	61.1	2009	L9^a,b^	Eu11
North America				
Fairbanks, AK, USA	64.9	2013	FA13 F3^b^	Am1
Honolulu Creek, AK, USA	64.1	2013	Ho13 F4^b^	Am2
Vancouver, BC, Canada	49.1	2003	Can3F20	Am3
Vancouver, BC, Canada	49.1	2003	Can3F24	Am4
Vancouver, BC, Canada	49.1	2003	Can3 F9	Am5
Ashford, WA, USA	46.8	2013	1ASH^b^	Am6
Grand Teton, WY, USA	43.4	1947	1510-1021.16	Am7
Cache County, UT, USA	41.7	1999	BS-3	Am8
Cache County, UT, USA	41.7	1999	BS-11	Am9
Cache County, UT, USA	41.7	1999	1595	Am10
Verdi, NV, USA	39.5	1949	15010-1021.17	Am11
Crested Butte, CO, USA	38.9	2003	C3 F2	Am12
Asia				
Kamchatka, Russia	56.2	2013	KR1323^b^	As1
Kamchatka, Russia	56.2	2013	KR1324^b^	As2
Kamchatka, Russia	56.2	2013	KR1309	As3
Kawasaki, Japan	34.8	—	1510-1021.13	As4
Kawasaki, Japan	34.8	1969	1263/20	As5
Selection experiment				
Oulanka, Finland	66.4	2013	Control 1^c^	Cont 1
Oulanka, Finland	66.4	2013	Control 2^c^	Cont 2
Oulanka, Finland	66.4	2013	Control 3^c^	Cont 3
Oulanka, Finland	66.4	2013	Selection 1^c^	Sel 1
Oulanka, Finland	66.4	2013	Selection 2^c^	Sel 2
Oulanka, Finland	66.4	2013	Selection 3^c^	Sel 3

Abbreviations: CDL = critical day length; NH = Nanda-Hamner. All raw data in the present study have been made and analyzed in Oulu. We have utilized here also some data that have been partially published in our earlier studies (see Suppl. Tables S2 and S3), applying slightly different statistical methods. ^a^CDLs estimated by Lankinen et al. (2013), ^b^CDLs estimated by Tyukmaeva et al. (2020) and ^c^CDLs and NH responses estimated by Kauranen et al. (2019). CDLs and/or NH responses of some strains have been estimated also in two other independent studies. ^d^CDLs studied by Tuykmaeva et al. (2011) and ^e^NH responses studied by Kauranen et al. (2013).

The study material also includes control and selection lines (3 replicates per line) from our previous quasinatural selection experiment for shorter CDL (Kauranen et al., 2019; see Table 1). This selection was started in Jyväskylä University from a large mass-bred population established from the progenies of > 100 fertilized females collected in Oulanka, Finland (Europe 66.40°N), and continued for 8 generations. Control and selection line replicates were maintained in Oulu University at 16°C in LL and LD 15:9, respectively, for 8-9 months (about 4 generations) after finishing active selection, using the same rearing methods are explained for the cline strains. The CDLs and the mean NH responses of the control and selection lines have been published by Kauranen et al. (2019) in 5 LDs and in continuous darkness (DD). Here we estimated the CDLs of all control and selection line replicates after collecting new data from two LD cycles (see Suppl. Table S2). We also measured the NH responses separately for each line replicate in the 5 LDs used in Kauranen et al. (2019), as well as in one new photoperiod (LD 12:4; see Suppl. Table S3).

### Experimental Procedures

**Figure 2.**
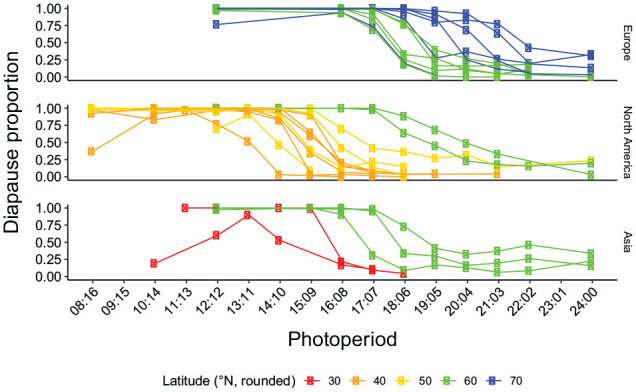
Photoperiodic response curves (PPRCs; proportion of diapausing females in different 24 h LDs) at 16°C for *D. montana* strains from different latitudes. Abbreviation: LD = Light:Dark.

CDLs of *D. montana* cline strains, as well as those of the control and selection line replicates, were estimated from the PPRCs based on female diapause proportions in several 24 h photoperiods between LD 8:16 and LD 24:0 (see Figures 2 and 6). In addition, female diapause proportions were measured in circadian and non-circadian photoperiods comprising of 12 h light 4 to 72 h darkness (total photoperiod varied from 16 to 84 h) and total darkness (DD) in NH experiments. The 6 LD cycles used in NH experiment were chosen on the basis of an earlier NH experiment on *D. montana* (Kauranen et al., 2013). The two most important LDs in the light of NH response were LD 12:24 (1.5 days) and LD 12:36 (2 days); in the “positive” NH resonance the latter LD should induce higher diapause (see Figure 1). Estimating the CDLs for most of the cline strains and all line replicates simultaneously and in the same chambers with NH experiments confirmed the internal coherence of the results and enabled us to trace the connection between CNL (24 h - CDL) and diapause induction under non-circadian conditions with a greater accuracy.

**Figure 3.**
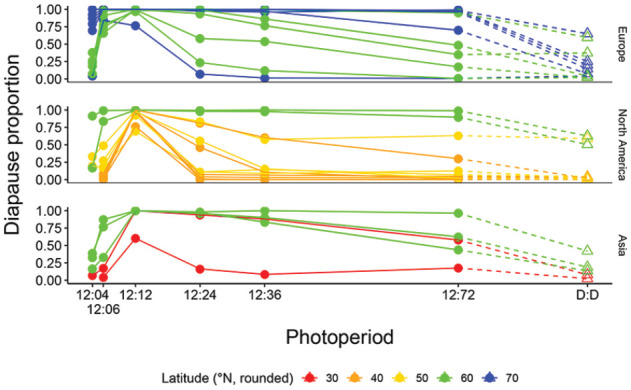
Proportion of diapausing females at 16°C in photoperiods with a day length of 12 h and a night length from 4 to 72 h and in continuous darkness (DD) in *D. montana* strains from different latitudes.

**Figure 4.**
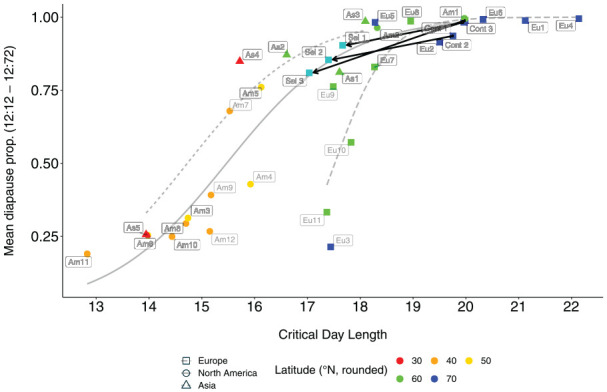
Average proportion of diapausing females at 16°C in photoperiods with a day length of 12 h and a night length from 12 to 72 h in NH experiment (Y axis) and the CDL (X axis) in *D. montana* strains from different latitudes and in the control and selection line replicates. Arrows between the control and selection line replicates indicate changes induced by selection for shorter CDL (explained in section 3.2.). Abbreviations: NH = Nanda-Hamner; CDL = critical day length.

**Figure 5.**
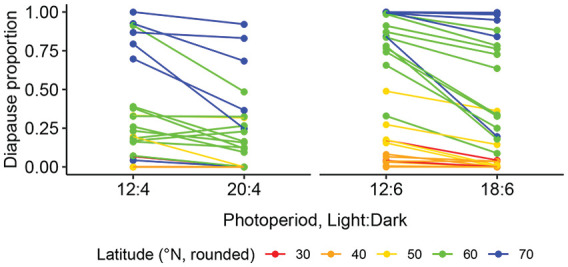
Female diapause proportions at 16°C in NH experiments and in 24 h LDs with the same night length (LD 12:4 vs LD 20:4 and LD 12:6 vs LD 18:6) for *D. montana* cline strains. Abbreviations: NH = Nanda-Hamner; LD = Light:Dark.

**Figure 6.**
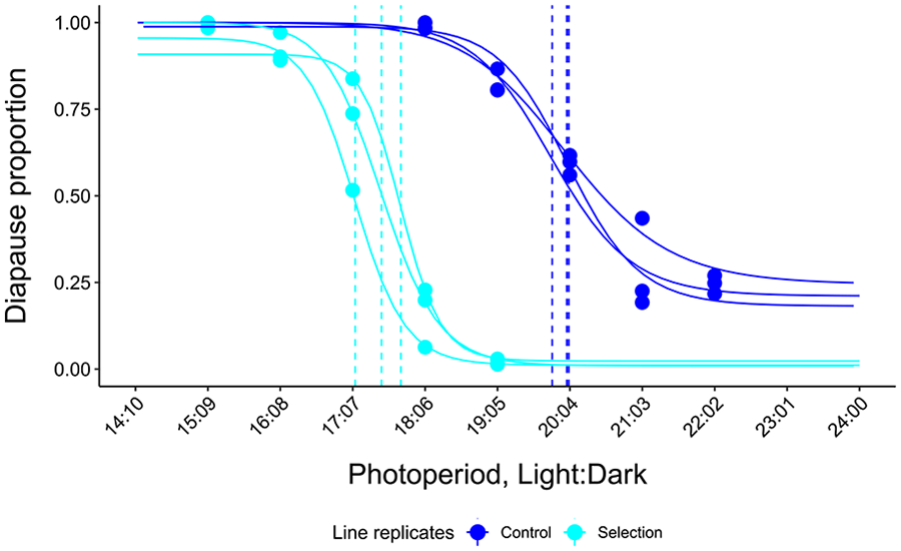
Photoperiodic response curves (PPRCs; proportion of diapausing females under different 24 h LDs) for the control and selection line replicates at 16°C. Abbreviation: LD = Light:Dark.

All experiments were performed at Oulu University in wooden or plastic, light-insulated and actively ventilated boxes, which were kept in the same temperature-controlled room at 16°C ± 0.3°C. The boxes were illuminated with one white fluorescent lamp per chamber (9 W, Megaman, Germany) with the light intensity of 300-1000 lux (corresponds about 5-16 W/m^2^). Samples of flies were transferred into one of the experimental photoperiods in malt media bottles as pupae. Emerging females and males were maintained together in these conditions until the females reached sexual maturity or entered reproductive diapause. The reproductive state of ether-anesthetized females was determined at the age of 21 ± 1 days by dissecting their ovaries under a light microscope. Females were considered as diapausing, if their ovaries were small and transparent and/or if they contained only a little yolk, or sexually mature, if their ovaries contained fully developed eggs. Sample sizes are given in Supplementary Tables S2 and S3. The total number of dissected females was about 105 000, including the data published in Lankinen et al. (2013), Kauranen et al. (2019) and Tyukmaeva et al. (2020).

### Statistical Analyses

Photoperiodic response curves (PPRCs) were estimated for each cline strain/line replicate with function drm (using option *type* *=* *“binomial”*) from R package drc (v3.0-1; Ritz et al., 2015) using a four-parameter log-logistic model:

$$y=c+\frac{d-c}{1+exp\left(b*\left(log\left(x\right)-log\left(CDL\right)\right)\right)}$$

*y* is the proportion of diapausing females; *x* is the number of light hours per day; *c* and *d* are the lower and upper limits (bound between 0 and 1), when *x* approaches 0 and 24, respectively, and *b* is the slope around CDL (the point of inflection of the PPRC; bound between 0.001 and 100000); and CDL is the critical day length defined as the dosage of light hours per day inducing reproductive diapause for 50% of the females (bound between 0 and 24).

While measuring female diapause proportions across photoperiods, we detected some abnormally low values at short day length in a few strains that are not exposed to such day lengths in their natural habitat. As these unexpected observations led to particularly high, and unrealistic, values of CDL, we decided to exclude them from our estimations. To do so, we first identified for each strain the maximum diapause proportion and its corresponding photoperiod and then removed any diapause proportions with a value below 90% of this maximum measured at a shorter photoperiod. This led to the removal of 1 data point for Eu3, Am6, Am11 and Am12, and 2 data points for As5 (see Suppl. Fig. S2).

To assess the effect of latitude on the CDL of cline strains, we fitted the CDL values of each strain to their approximate latitude of origin using the function glm (package stats) with a quasibinomial model and a logit link function. The models were tested with and without the continental provenance included as covariate. Moreover, after calculating the mean diapause proportions of these strains in ≥ 24 h photoperiods (12:12, 12:24, 12:36, and 12:72) in NH experiment, we tested the effect of latitude to this trait using function glm (package stats) with a quasibinomial model and a logit link function. These models were also tested with and without the continental provenance as a covariate.

To assess the effect of extra short nights on females’ diapause proportions in *D. montana* cline strains in unnatural and natural photoperiods (12:4 vs 20:4 and 12:6 vs 18:6, respectively), we ran three quasibinomial models with a logit function. In the first model, we fitted the diapause proportions measured in 12:4, 20:4, 12:6, and 18:6 to the hours of light and darkness, to the CDL of each strain and to the ratio of light and darkness. In the second and third models, we fitted female diapause proportions at 12:4 and 20:4, and 12:6 and 20:6, respectively, to the hours of light and the CDL of each strain.

We next performed pairwise comparisons between the CDLs of each selection and control line replicate using the function EDcomp (package drc; standard errors, confidence intervals and t-values computed using the “delta” intervals), and applying a Bonferroni correction for 15 comparisons. We also used a binomial model and a logit link function to test whether the previous selection for shorter CDL (Kauranen et al., 2019), and the maintenance of the selection line replicates under LD 15:9, had induced changes in lines’ mean diapause proportions in ≥ 24 h photoperiods in NH experiment. Here we converted the developing and non-developing females as binary values, respectively 0 and 1, as a response variable and the experimental status (control or selection line), photoperiod (LDs 12:12, 12:24, 12:36, and 12:72) and line identity (replicate number) as observed variables.

Finally, we used a quasibinomial model and a logit link function to test the effect of the CDL and the latitudinal origin of each strain on the mean diapause proportions of given strains in ≥ 24 h photoperiods. Moreover, we tested if the selection toward lower CDLs had changed the mean diapause proportion of the experimental lines in these photoperiods the same way as in the cline strains.

Finally, we used a quasibinomial model and a logit link function to test the effects of CDL on the mean diapause proportions in ≥ 24 h photoperiods in the combined data sets for the cline strains and the experimental lines, using the region of origin as covariate. To test if the relationship between these traits is homogeneous between cline strains and experimental lines, we compared the fit of the model with and without the experimental status of the strains/lines as an additional covariate to the model: cline strains on one hand and control and selection lines on the other.

All statistical analyses were performed using R version 3.4.4 (R Core Team, 2018), and all plots were done using R package ggplot2 (v 3.3.0; Wickham, 2016).

## Results

The goal of the present studies was to disentangle the mechanisms driving and maintaining latitudinal variation in the function of PPTM that underlays *D. montana* females’ reproductive diapause, and to find out whether this variation is based on qualitative or quantitative time-measurement system. By performing studies on *D. montana* strains originating from different latitudes and on the selection and control lines from our earlier selection experiment for shorter CDL (Kauranen et al., 2019), we were able to trace the effects of selection on PPTM both in natural and artificial environment.

### Latitudinal Variation in *D. Montana* Strains’ CDL and Female Diapause Proportions in Natural 24 h Cycles and Artificial Environments of Non-24 h Cycles

#### Latitudinal Variation in CDL

Latitudinal variation in CDL was evident on all continents, PPRCs of the strains from the high latitudes (above 55°N) showing over 50% diapause proportions at much longer day lengths than those of the more southern strains (Figure 2). The CDLs of the northernmost strains from Finland (Europe), Alaska, USA (North America), and Kamchatka, Russia (Asia) varied between LDs 17:7 and 22:2. Among southern strains, the shortest CDLs belonged to North American strains from Nevada and Utah, USA (latitude 40°N) and to Asian strains from Japan (30°N). The CDLs of individual strains are given in Supplementary Table S1 and the PPRCs, where they are based on, in Supplementary Fig. S2A. Overall, CDL showed a significant correlation with latitude (*r* = 0.17408; *p*-value = 9.30 × 10^-6^; Suppl. Fig. S5), with no effect of the continental variable (at alpha = 0.05). Interestingly, the time spent in lab conditions had no impact at all on this trait, as even over 70 years old lab strains showed similar phenotypes as other strains sampled more recently at the same latitude.

#### Latitudinal Variation in Female Diapause Proportions Under Extra Long and Short Photoperiods

In NH experiments, all *D. montana* strains had at most one high diapause peak (in LD 12:12), and none of them showed cyclic variation in female diapause proportions at 24 h intervals, not even a slight increase in LDs 12:36. In nearly all strains originating above the latitude of 65°N, female diapause proportion remained close to 100% under all LDs and decreased below 50 % only in DD (Figure 3; Suppl. Fig. S3A; Suppl. Table S1). Other northern strains showed high variation in female diapause proportions both in the extra long and extra short photoperiods, and in the southernmost strains the diapause proportions were below 50% in all LDs except LD 12:12. In DD, variation between the northern strains was quite large, while in all southern strains, except one, the diapause proportions dropped in darkness below 50 % (DD; see Figure 2; Suppl. Table S1).

The logistic regression of the strains’ mean diapause proportions in ≥ 24 h photoperiods showed a significant correlation with latitude (beta = 0.12, *p*-value = 0.00447; Suppl. Fig. S6), with no effect of the continental factor (at alpha = 0.05). Moreover, comparison between the mean diapause proportions in these photoperiods and the CDLs of the same strains (see Figure 4) revealed a significant correlation between these traits in Europe (beta = 1.975, *p*-value = 0.0183) and North America (beta = 0.888, *p-*value = 0.000253), while in Asia the correlation remained only close to significance (beta = 0.907, *p*-value = 0.0625). The slope when including all continents was highly significant (beta = 0.654, *p*-value = 6.58 × 10^-6^). The Finnish strains Eu3, Eu10, and Eu11 had about the same CDL as the other northern strains, but their mean diapause percentages in ≥ 24 h photoperiods resembled those of the southern strains, which suggests that the correlation between these traits is not total.

In NH experiments performed in extra short nights (LDs 12:4 and 12:6), the dark periods could be too short for diapause induction, but at the same time females experienced a higher number of Light:Dark cycles than in LD 12:12. In these photoperiods, the females of the northern strains entered diapause even when the night length was shorter than their CNL, while the diapause proportions of all southern strains remained quite low (Figure 3; Suppl. Table S1). In the combined data set for all strains, long CDL and a higher number of dark hours (6 or 4 h) per photoperiod increased female diapause proportion significantly (respective *p*-values < 2 × 10^-16^and 0.00087; Suppl. Table S4), while a higher number of light hours (12, 18, or 20 h) decreased it (*p*-value = 0.010; Suppl. Table S4), and the ratio of light and dark hours had no significant effect on it (*p*-value = 0.107; Suppl. Table S4). Comparing the strains’ diapause proportions in extra short photoperiods versus natural 24 h cycles with the same night length (LD 12:4 vs 20:4 and LD 12:6 vs 18:6; see Figure 5) showed the same trends. Here the effects of the number of light hours and the CDL on diapause proportions were significant (*p*-values for light hours and CDL in the first-mentioned comparison 0.00543 and 1.50 × 10^-12^ and in the latter ones 2.00 × 10^-6^ and 4.92 × 10^-16^, respectively; Suppl. Table S4).

### Connection Between the CDL and the Female Diapause Proportions in NH Experiments in the Selection and Control Lines

#### Differences Between the Control and Selection Lines in CDL

CDLs estimated from the PPRCs of the control and selection line replicates varied between LDs 19.8:4.2 and 20.1:3.9 for the control line replicates and between LDs 17.0:7.0 and 17.6:6.4 for the selection line ones (Figure 6; Suppl. Table S1; Fig. S2B,). CDLs of the control line replicates resembled those of the other strains (Eu1 and Eu2) from the flies’ home population in Oulanka, Finland (66.40°N), while those of the selection line replicates were of the same level as that of the strains Eu9, Eu10 and Eu11 from the southern Finland (61.10°N). Pairwise comparison of the CDL estimates showed the CDLs of all line replicates, except the control line ones, to differ significantly from each other (*p*-values < 0.005) even after Bonferroni correction for 15 comparisons (Suppl. Table S5).

#### Differences Between the Control and Selection Lines in Female Diapause Proportions in NH Experiments

**Figure 7.**
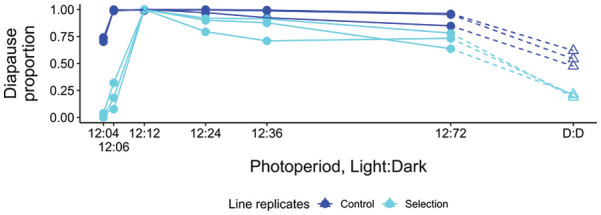
Proportion of diapausing females in different photoperiods with a day length of 12 h and a night length from 4 to 72 h, and in continuous darkness (DD) in the control and selection line replicates at 16°C. The data for LD 12:4 is produced in the present study; all other data iares from Kauranen et al. (2019). Abbreviation: LD = Light:Dark.

To find out whether selection for shorter CDL had induced changes in female diapause proportions in ≥ 24 h photoperiods in NH experiment, we tested the effects of the experimental status (control or selection line), LD and line identify (replicate number) on the mean diapause proportions at LD 12:12, 12:24, 12:36, and 12:72 using a binomial model (with a logit link function). Overall, all three factors had a significant effect on female diapause proportion (Residual deviances equal to 484.06, 155.23, and 66.21, respectively, and all *p*-values below 2.2 x 10^-16^). Under the retained model, the diapause proportions of the selection line replicates were slightly lower than those of the control line replicates, with a significant effect of the experimental status (estimate = -2.67, std. error = 0.23, *p*-value < 2.2 x 10^-16^), LD (estimates between − 4.4 and 5.6, std. error between 1.003 and 1.007, and *p*-values all below 1 x 10^-4^) and line identity (although only for the lines Control 2, Selection 1, and Selection 2, with estimates between -1.3 and 0.8, std. error between 0.11 and 0.36 and *p*-values below 0.05; Figure 7; Suppl. Table S3).

As illustrated by the arrows in Figure 4, selection for shorter CDL had changed the correlation between CDL and the mean diapause proportion in ≥ 24 h photoperiods in selection vs control lines, but the change was smaller than could be expected on the basis of respective data for *D. montana* cline strains. To test whether the difference between the control and selection lines was statistically significant, we used a quasibinomial model with the mean diapause proportion in ≥ 24 h photoperiods as response variable and the CDL and region as the observed variables. Moreover, we used an analysis of deviance directed on the combined data set of the cline strains and the control and selection lines to test whether adding the experimental status to the model would result in a better model. Doing so resulted in a significantly better model (with the residuals of deviance with and without including the experimental status at 2.664 and 3.553 respectively, and p-value = 0.0019 using a chi² test), with a significant effect of the experimental status of the strains (estimate = -0.176, std error = 0.081, *p*-value = 0.04), as was the effect of CDL (estimate = 0.124, std error = 0.017, *p*-value = 5.1 x 10^-8^), but not the region of origin (at alpha = 0.05). In other words, selection for shorter CDL had induced similar differences between the control and selection lines as detected between *D. montana* cline populations.

In LD 12:4, the diapause proportions of the control line replicates remained in 75%, while those of the selection line replicates were close to 0 (Figure 7). These proportions were slightly lower than the respective proportions in LD 12:6 (Figure 7; Suppl. Table S1).

## Discussion

Insect species with a wide distribution offer a great opportunity to trace latitudinal variation in traits that are important in reproduction and stress tolerances, and particularly in the contribution of circadian clock(s) and/or PPTM in their regulation. Our study species, *D. montana*, shows steep latitudinal clines in CDL and its temperature-sensitivity (Tyukmaeva et al., 2020), as well as in the flies’ basal cold tolerance (Wiberg et al., 2020). In the present study, variation in the diapause proportions of *D. montana* cline strains, and the control and selection lines, in extra long and short photoperiods revealed many interesting aspects of PPTM related to our study questions. First, none of the strains showed signs of circadian rhythmicity in their mean diapause proportions in ≥ 24 h photoperiods in NH experiments, and females of the northern strains possessed higher stability against perturbations in the photoperiod length than those of the southern strains. Second, latitudinal variation in strains’ mean diapause proportions in ≥ 24 h photoperiods showed correlation with their CDL. Third, the high diapause proportions of the northern strains in extra long and short photoperiods suggest that the photoperiodic clock measures night length quantitatively and that both the accumulation of a high number of short nights or a low number of long nights at counter level induces diapause in these strains. Together these findings suggest that the function of PPTM of *D. montana* can best be explained by the quantitative versions of the damped external coincidence model.

Earlier studies on insect species with a “negative NH response” have shown that the species may display a rapid increase in diapause incidence, when the night length exceeds their CNL, and then achieve a high non-rhythmic plateau in the prolonged photoperiods (Vaz Nunes and Saunders, 1999; Saunders et al., 2004; Wang et al., 2004; Huang et al., 2005), or they may show a high diapause peak in LD 12:12 after which their diapause proportion decreases toward longer nights (Adams, 1986). Most *D. montana* strains from high latitudes showed close to 100 % diapause at 16°C in all ≥ 24 photoperiods, while the rest of the strains had a high diapause peak in LD 12:12 after which their diapause proportions decreased gradually toward longer photoperiods. This, and the fact that the females of some northern strains enter diapause even in continuous darkness, gives support to Pittendrigh’s (1972) argument that a high diapause incidence of northern strains under prolonged nights could refer to a high stability of female diapause response against perturbations in the photoperiod length. Lumme and Oikarinen (1977) has reported an increase in female diapause proportions in darkness toward high latitudes also in another *virilis* group species, *Drosophila littoralis*. However, the type of negative NH response (Vaz Nunes and Saunders, 1999), as well as the females’ tendency to mate in DD, (Saunders, 2002), may vary according to the temperature, and thus also *D. montana* females of the southern populations could enter diapause under ≥ 24 photoperiods and DD in a lower temperature.

Strong correlation detected between CDL and the mean diapause proportion in ≥ 24 photoperiods in *D. montana* strains suggests that latitudinally varying selection pressures have either driven the traits into the same direction, or that the selection for shorter or longer CDL has affected also female diapause incidence under non-circadian photoperiods. A similar correlation, detected between the control and selection line replicates, gives support to the latter option and indicates at least partial causal connection between these traits. However, correlation between CDL and the mean diapause proportions in ≥ 24 photoperiods may not be total, as shown by the behavior of strain Eu3 in NH experiment. This strain resembled other northern strains by its long CDL and high diapause incidence in LD 12:6, while its mean diapause proportion in ≥ 24 h photoperiods was as low as in the most southern strains.

Measuring and/or accumulation of long and short night cycles may occur through different mechanisms at a clock or at a counter level. For example, the photoperiodic clock of *Megoura viciae* vetch aphids consists of a slowly damping long-night system and a rapidly damping short-night system and that of *Aphis fabae* black bean aphids of a rapidly damping long-night system and a damping or self-sustained short-night system (Hardie and Vaz Nunes, 2001). Moreover, in both of these species (Hardie and Vaz Nunes, 2001), as well as in *S. argyrostoma* flesh flies (Saunders, 1992), the long-night accumulation is temperature compensated, whereas the short-night accumulation is not. Lack of NH rhythms in *D. montana* prevented us from tracing latitudinal variation in the damping of these rhythms under the long and short photoperiods, but the diapause behavior of the northern strains of this species gave interesting information on other aspects of PPTM. Females of these strains entered diapause in extra short photoperiods, where the length of the dark period could be shorter than their CNL, but where the females experienced a higher number of photoperiodic cycles than in 24 h photoperiod (up to 12 cycles vs 8 cycles in 24 h photoperiod). Most likely explanation for this is that the high number of daily cycles accumulated in the photoperiodic counter compensated the shortness of night. On the other hand, females entered diapause also in extra long photoperiods, which shows that long nights can evoke diapause even if the cycle number remains low (in longest photoperiods only ~3 cycles). All this suggests that the photoperiodic clock of *D. montana* females measures night length quantitatively and that the role of photoperiodic counter varies according to the length of the photoperiod.

The data accumulating on *D. montana* raise a question on which clock/timer models can best explain the function of PPTM in this species. An hourglass model could explain female diapause behavior under extra short nights, but it cannot explain why the females of northern *D. montana* strains enter diapause in prolonged nights, some even in DD, and why there is such a high latitudinal variation in female diapause incidence in extra long nights. This is because an hourglass executes only a single act of time measurement during the prolonged nights, and it requires a period of illumination after each night to sustain the mechanism. Internal coincidence model (Pittendrigh, 1972) cannot explain our results either, as *D. montana* flies lack the morning oscillators and cannot measure seasonal changes in day length from the phase relationship between the morning and evening oscillators (Kauranen et al., 2012). The basic and damped versions of the external coincidence model assume that variation in insect NH response is based on variation in the period of oscillation of the photoinducible phase at the end of 24 h cycles, and the latter version also suggests that this oscillation is damped under prolonged darkness (Vaz Nunes and Saunders, 1999). These models cannot explain the high diapause proportions of northern *D. montana* strains in extra short nights, and the fact that nearly all *D. montana* strains with a high diapause peak in LD 12:12 had a higher diapause proportion in LD 12:24 than in LD 12:36 (multiple of 24 h) indicates that the possible circadian oscillations should be rapidly damping. The quantitative versions of the damped external coincidence model give the most feasible explanation to our results. In these models, insects are expected to synthesize hypothetical “diapause titer” during the long photoinducible phases occurring at night, and accumulate it daily in the counter system, so that a photoperiodic response is initiated when the substance reaches an internal reference threshold (Saunders, 1981; Tagaya et al., 2010; Meuti and Denlinger, 2013; Goto, 2013). Light periods play an important role in both regulating the titer quantity and in accumulating it in the counter system, while population differences in diapause proportions in specific photoperiods can be induced by variations of a diapause threshold, specific to each female, and/or of the synthesis or degradation rates of the diapause titer (Tagaya et al., 2010). According to these models, latitudinal variation in *D. montana* CDL and NH responses could be explained by females of the northern strains synthesizing hypothetical diapause titer more efficiently during the dark period and/or these females having a lower diapause threshold than those of the southern strains. The fact that females of northern strains in some cases entered diapause in DD suggests that in *D. montana* the substrate is synthesized constitutively during darkness, and that its accumulation in the counter system does not necessarily require a light period.

Several timer models suggest that the function of PPTM involves a specific substrate “diapause titer” synthesized during the dark periods (Lees, 1973; Saunders, 1981; Tagaya et al., 2010). But what is this titer, and does its synthesis require several steps from the night length measurement to photoperiodic counter? According to Meuti and Denlinger (2013), the dominant endocrine mechanism that halts ovary development is a failure of the brain and its associated endocrine organs to produce the hormones that trigger maturation of reproductive organs. These authors suggest that neuropeptide pigment-dispersing factor (PDF), which is expressed in many central circadian clock cells and is an important output of circadian clocks in *D. melanogaster*, could accumulate under successive short nights and induce diapause when reaching a critical threshold. Contrary to *D. melanogaster, virilis* group species lack PDF in their short ventrolateral clock neurons (s-LNvs), but express it in the central brain (Bahn et al., 2009, Kauranen et al., 2012, Hermann et al., 2013), which raises a question on whether PDF, or the lack of it, in specific neurons could explain species differences in diapause induction. Moreover, Veerman (2001) has suggested that diapause induction may ultimately consist of the photoperiod-dependent synthesis (and further processing) of one or more transcription factors that regulate the expression of diapause-related genes. Kauranen et al. (2019) have listed 16 gene clusters, including the ones involved in signal transduction, membrane properties, immunologlobulins and development, which have diverged significantly during the selection for shorter CDL in *D. montana*, and Salminen et al. (2015), and Kankare et al. (2016) have identified genes that show expression changes at different phases of diapause in this species. Maybe, a closer look at the function of these genes in northern and southern *D. montana* strains under circadian and non-circadian photoperiods could help to find good candidates for a diapause titer.

## Conclusions

In most arthropod species, induction of photoperiodic diapause is an active process that requires counts of short days, while ovarian maturation itself is a default state proceeding unless environmental cues are halting it (Saunders, 2002; Hua et al., 2005). This is also true for *D. montana* females, which develop ovaries in continuous light (Kauranen et al., 2016) and in 24 h LDs where the night length is shorter than their CNL (Tyukmaeva et al., 2020). In the present study, a few northern strains of this species had high diapause proportions even under DD, which raises the question of whether the light period has lost its importance in these strains. This study also revealed several other features of PPTM that may have helped the flies to adapt to seasonally varying environments at high latitudes. These features include quantitative measurement of night lengths, flexibility of CNL in extra short photoperiods and differences in the accumulation short and long night information at counter level.

Saunders (2002) has stated that the most inductive short night cycles for diapause induction are often the ones whose LD cycle is close to 24 h, and Pittendrigh (1972) has argued that a high diapause incidence of northern insect populations under prolonged nights refers to a high stability of their diapause response against perturbations in the photoperiod length. Our study gives support to both of these arguments. Most *D. montana* strains showed high diapause only in LD 12:12, and their diapause proportions decreased close to zero under the longer photoperiods. On the other hand, the northernmost *D. montana* strains had close to 100 % diapause under all combinations of night lengths from 12 to 72 h and LD cycles from 21 to 6, which shows that these strains are resistant to deviations from 24 h cycle.

Among the presented models, a quantitative version of the damped external coincidence model could best explain our results as it offers a possibility to flexible responses to prevailing environmental conditions. However, there are still several open questions that need to be addressed in order to understand the function of PPTM in diapause induction. We are presently performing NH experiments for *D. montana* strains at different temperatures to trace the effects of temperature on latitudinal variation in female diapause responses in extra long and short photoperiods. We also aim to perform “mirror” NH experiments with a constant night length and varying day lengths to trace latitudinal variation in the role of the light period on female diapause induction. These studies, combined with genetic and neurological studies, should bring more information on the function of PPTM in northern insect species.

## Supplemental Material

sj-pdf-1-jbr-10.1177_0748730421997265 – Supplemental material for Nanda-Hamner Curves Show Huge Latitudinal Variation but No Circadian Components in Drosophila Montana Photoperiodism Click here for additional data file. Supplemental material, sj-pdf-1-jbr-10.1177_0748730421997265 for Nanda-Hamner Curves Show Huge Latitudinal Variation but No Circadian Components in Drosophila Montana Photoperiodism by Pekka Lankinen, Chedly Kastally and Anneli Hoikkala in Journal of Biological Rhythms
