# Supplementary material for: Nanda-Hamner Curves Show Huge Latitudinal Variation but No Circadian Components in Drosophila Montana Photoperiodism
Source: J Biol Rhythms. 2021 Mar 22;36(3):226–38. doi: 10.1177/0748730421997265 (PMC8114436; doi:10.1177/0748730421997265)
Supplement: sj-pdf-1-jbr-10.1177_0748730421997265 – Supplemental material for Nanda-Hamner Curves Show Huge Latitudinal Variation but No Circadian Components in Drosophila Montana Photoperiodism [file sj-pdf-1-jbr-10.1177_0748730421997265.pdf]

SUPPLEMENTARY MATERIAL

Table S1. The codes, latitudes, CDLs and NH scores (average diapause proportions at 16ºC in LDs 12:12, 12:24, 12:36 and 12:72) for *D. montana* strains and selection experiment lines. Female diapause proportions are given for each strain / line in continuous darkness (DD), in the night lengths of 4, 6, 12, 24, 36 and 72 h in NH experiment and in the night lengths of 4, 6 and 12 h in 24 h cycles in the data used in CDL estimations.

**Strain code**	**Latitude**	**CDL**	**NH score**	**Diapause proportion**									
				**NH D:D**	**CDL 20:4**	**NH 12:4**	**CDL 18:6**	**NH 12:6**	**CDL 12:12**	**NH 12:12**	**CDL 12:24**	**NH 12:36**	**CDL 12:72**
**Europe**													
Eu1	66.4	21.12	0.99	0.14	0.92	1.00	0.99	1.00	1.00	1.00	0.99	1.00	0.96
Eu2	66.4	19.50	0.91	0.07	0.25	0.79	1.00	0.99	0.99	0.99	0.99	0.97	0.70
Eu3	65.7	17.44	0.21	0.04	NA	0.04	0.20	0.84	NA	0.77	0.07	0.01	0.01
Eu4	65.7	22.14	1.00	0.20	0.83	0.87	0.95	0.99	1.00	1.00	1.00	1.00	0.98
Eu5	65.7	18.28	0.98	0.25	0.37	0.70	0.84	1.00	1.00	1.00	0.98	0.96	0.99
Eu6	65.4	20.32	0.99	0.65	0.68	0.93	0.98	1.00	1.00	1.00	0.99	0.99	0.98
Eu7	64.3	18.27	0.83	0.04	0.16	0.23	0.78	0.99	0.98	0.98	0.99	0.86	0.48
Eu8	61.1	18.95	0.99	0.60	0.27	0.18	0.76	0.91	1.00	1.00	1.00	0.99	0.95
Eu9	61.1	17.49	0.76	0.37	0.12	0.38	0.25	0.66	1.00	1.00	0.94	0.77	0.35
Eu10	61.1	17.83	0.57	0.02	0.10	0.26	0.33	0.74	0.98	0.99	0.58	0.54	0.17
Eu11	61.1	17.37	0.33	0.02	0.00	0.07	0.18	0.78	0.97	0.97	0.24	0.12	0.01
**North America**													
Am1	64.9	19.97	1.00	0.63	0.49	0.91	0.88	0.99	1.00	1.00	1.00	1.00	0.99
Am2	64.1	18.32	0.96	0.50	0.23	0.17	0.64	0.84	1.00	1.00	0.98	0.98	0.90
Am3	49.1	14.74	0.31	0.01	NA	NA	0.14	0.27	0.95	0.93	0.11	0.09	0.13
Am4	49.1	15.92	0.43	0.01	NA	0.20	0.01	0.18	0.98	0.98	0.56	0.16	0.02
Am5	49.1	16.12	0.76	0.59	0.32	0.33	0.36	0.49	1.00	1.00	0.84	0.58	0.63
Am6	46.8	13.98	0.25	0.05	NA	NA	NA	0.16	NA	0.70	0.11	0.15	0.07
Am7	43.4	15.53	0.68	0.02	NA	NA	0.04	0.04	1.00	1.00	0.81	0.60	0.30
Am8	41.7	14.70	0.29	0.04	NA	NA	NA	0.08	0.99	0.99	0.07	0.06	0.05
Am9	41.7	15.17	0.39	0.02	NA	NA	0.04	0.07	0.99	0.99	0.46	0.11	0.01
Am10	41.7	14.44	0.25	0.00	NA	NA	0.00	0.00	0.98	0.98	0.01	0.00	0.01
Am11	39.5	12.83	0.19	0.01	NA	NA	NA	0.01	0.76	0.76	0.00	0.00	0.00

Am12	38.9	15.15	0.27	0.03	NA	NA	NA	0.03	0.97	0.97	0.04	0.03	0.03
**Asia**													
As1	56.2	17.61	0.81	0.14	0.16	0.39	0.34	0.77	1.00	1.00	0.98	0.84	0.44
As2	56.2	16.61	0.87	0.19	0.13	0.16	0.09	0.33	0.97	1.00	0.96	0.90	0.62
As3	56.2	18.10	0.99	0.42	0.32	0.33	0.73	0.87	1.00	1.00	0.98	1.00	0.96
As4	34.8	15.72	0.85	0.08	NA	0.07	0.04	0.17	1.00	1.00	0.94	0.88	0.58
As5	34.8	13.94	0.26	0.02	NA	NA	NA	0.04	NA	0.60	0.16	0.08	0.18
**Selection experiment**													
Cont 1	66.4	19.96	0.98	0.54	0.60	0.73	1.00	1.00	NA	0.99	1.00	0.99	0.95
Cont 2	66.4	19.75	0.94	0.48	0.56	0.70	0.98	0.99	NA	1.00	0.97	0.92	0.85
Cont 3	66.4	19.98	0.99	0.62	0.62	0.74	0.98	0.99	NA	1.00	1.00	1.00	0.96
Sel 1	66.4	17.67	0.90	0.19	NA	0.04	0.23	0.32	NA	1.00	0.92	0.91	0.78
Sel 2	66.4	17.40	0.85	0.22	NA	0.00	0.20	0.18	NA	1.00	0.90	0.88	0.64
Sel 3	66.4	17.03	0.81	0.21	NA	0.03	0.06	0.08	NA	1.00	0.79	0.71	0.73

Table S2. Number of females (sample size) studied under different photoperiods for preparing photoperiodic response curves (PPRCs) and estimating CDL at 16ºC.

Strain	Sample size / photoperiod														
code	08:16	10:14	11:13	12:12	13:11	14:10	15:09	16:08	17:07	18:06	19:05	20:04	21:03	22:02	24:00
**Europe**															
Eu1				397				*96*		*176*	*131*	394	*257*	*121*	311
Eu2				165				66	116	285	307	344	169	156	
Eu3				235				204	412	551	*186*				
Eu4				305				*77*	*74*	117	*199*	202	*187*	*99*	212
Eu5				134					*152*	*101*	*170*	*120*	*239*	*131*	176
Eu6				123					*79*	*119*	*92*	196	*137*	86	150
Eu7				324				*99*	*144*	253	*102*	216		82	
Eu8				96				*81*	208	1031	335	377	*62*	92	26
Eu9				298				*86*	150	374	*85*	*110*	*93*	54	
Eu10				464				164	233	180	*107*	*126*	*41*	118	
Eu11				197				*122*	249	327	*64*	*57*	*45*		
**North America**															
Am1				178		224			111	348	266	484	264		255
Am2				128		61		42	117	236	187	297	56	103	86
Am3	100			916	307	294	337	695	295	319					
Am4	39			96		99	120	370	387	97					
Am5	46			273		106	117	1352	709	569	96	249	117		106
Am6				414	154	191	215								
Am7	39	198		397		159	313	245	128	308	46				
Am8		176		308	154	247	184	236	52						
Am9		45		176		79	321	319	189	54			24		
Am10		156		253	127	192	125	353	80	59					
Am11	277	147	141	421	167	195		48							
Am12	71	191		595		184	223	639	274						
**Asia**															
As1				114				*102*	*168*	*309*	*206*	*116*	*132*	240	240
As2				114			171	264	392	292	136	*151*	127	165	206
As3				67				63	220	818	249	607	129	27	434
As4			32	265		180	308	210	153	276					
As5		228		234	88	150		190	38						
**Selection experiment**															
Cont 1				133						*181*	*257*	*289*	*319*	*296*	
Cont 2				160						*331*	*335*	*491*	*338*	*479*	
Cont 3				87						*172*	*187*	*313*	*171*	*399*	
Sel 1				130			67	*313*	*303*	*223*	*138*				
Sel 2				161			155	*247*	*332*	*282*	*143*				
Sel 3				221			48	*355*	*494*	*507*	*131*				

The data marked with italics have been published in Lankinen et al. (2013), Kauranen et al. (2019) or Tuykmaeva et al. (2020). All other data are new or completed for the present study.

.

Table S3. Number of females (sample size) studied under different photoperiods and darkness (DD) for estimating female diapause proportions in NH experiments at 16°C.

	Sample sizes in NH photoperiods					
**Strain code**	12:04	12:06	12:24	12:36	12:72	DD
**Europe**						
Eu1	77	80	144	145	158	292
Eu2	306	199	262	261	249	203
Eu3	117	544	228	304	137	119
Eu4	244	149	103	45	257	166
Eu5	328	80	171	129	128	171
Eu6	241	166	163	307	260	371
Eu7	330	215	233	189	186	117
Eu8	190	427	187	683	640	225
Eu9	200	495	204	605	146	83
Eu10	119	358	432	654	581	104
Eu11	195	575	313	655	310	101
**North America**						
Am1	199	118	212	112	499	228
Am2	201	196	158	233	358	143
Am3		472	394	645	270	68
Am4	414	171	204	225	141	143
Am5	217	135	146	299	182	100
Am6		116	211	393	176	111
Am7		517	436	898	781	54
Am8		304	187	556	128	141
Am9		61	193	235	94	128
Am10		58	118	206	112	131
Am11		169	58	60	125	154
Am12		125	409	674	227	120
**Asia**						
As1	236	673	282	352	550	279
As2	233	511	173	121	530	154
As3	290	506	207	102	576	119
As4	148	539	228	716	261	151
As5		347	338	226	74	92
**Selection experiment**						
Cont 1	290	*167*	*228*	*257*	*353*	*269*
Cont 2	276	*371*	*388*	*198*	*159*	*328*
Cont 3	283	*252*	*242*	*224*	*318*	*221*
Sel 1	257	*250*	*250*	*359*	*293*	*155*
Sel 2	124	*280*	*193*	*270*	*372*	*223*
Sel 3	64	*248*	*239*	*314*	*368*	*341*

The data for LDs 12:06 – 12:72 and DD for selection experiment, marked with italics, have been published in Kauranen et al. (2019).

Table S4. GLM tests (quasibinomial model with a logit function) on the effects of 4 and 6 h nights on *D. montana* strains' diapause proportions in extra short photoperiods in NH experiment vs. natural 24 h photoperiods. Comparisons were made between all photoperiods (12:4. 20:4. 12:6 and 18:6), between LD 12:4 and 20:4 and between LD 12:6 and LD 18:6.

GLM test on extra short night (photoperiods 12:4, 20:4, 12:6 and 18:6).

	**Estimate**	**Std. Error**	**t-value**	**Pr(>|t|)**
**(Intercept)**	-25.60092	2.97484	-8.606	7.13E-14
**CDL**	1.09177	0.07918	13.788	< 2E-16
**Dark hours**	1.69193	0.49401	3.425	0.000873
**Light hours**	-0.43117	0.16539	-2.607	0.010436
**Ratio Light/Dark hours**	1.22014	0.75073	1.625	0.107048

GLM test on 4 hours nights (photoperiods 12:4 and 20:4).

	**Estimate**	**Std. Error**	**t-value**	**Pr(>|t|)**
**(Intercept)**	-15.79605	1.81337	-8.711	8.33E-12
**Light hours**	-0.11206	0.03864	-2.9	0.00543
**CDL**	0.91484	0.09959	9.186	1.5E-12

GLM test on 6 hours nights (photoperiods 12:6 and 18:6).

	**Estimate**	**Std. Error**	**t-value**	**Pr(>|t|)**
**(Intercept)**	-18.34018	1.78869	-10.253	3.48E-14
**Light hours**	-0.25951	0.04862	-5.337	2E-06
**CDL**	1.28735	0.11185	11.51	4.92E-16

Table S5. Pairwise comparisons of the CDLs between the selection and control line replicates.

Comparison	Estimate	Std error	t-value	p-value*
Sel 3/Cont 3	0,8529	0,0040	-37,12395769	**0,00000000**
Sel 2/Cont 3	0,8622	0,0040	-34,54962143	**0,00000000**
Sel 1/Cont 3	0,8720	0,0041	-31,52331422	**0,00000000**
Sel 3/Cont 2	0,8815	0,0041	-28,99081291	**0,00000000**
Sel 2/Cont 2	0,8847	0,0042	-27,33099084	**0,00000000**
Sel 1/Cont 2	0,8943	0,0042	-24,87583161	**0,00000000**
Sel 3/Cont 1	0,8546	0,0106	-13,77564653	**0,00000000**
Sel 2/Cont 1	0,8737	0,0108	-11,69856193	**0,00000000**
Sel 1/Cont 1	0,8864	0,0110	-10,33360176	**0,00000000**
Sel 3/Sel 1	0,9641	0,0040	-9,04074583	**0,00000000**
Sel 3/Sel 2	0,9781	0,0039	-5,60637930	**0,00000002**
Sel 2/Sel 1	0,9857	0,0041	-3,52177321	**0,00042867**
Cont 3/Cont 2	1,0109	0,0053	2,06905608	0,03854082
Cont 2/Cont 1	0,9912	0,0125	-0,70792518	0,47899172
Cont 3/Cont 1	1,0020	0,0126	0,15718919	0,87509574

* values in bold are significant after Bonferroni correction at alpha = 0.05

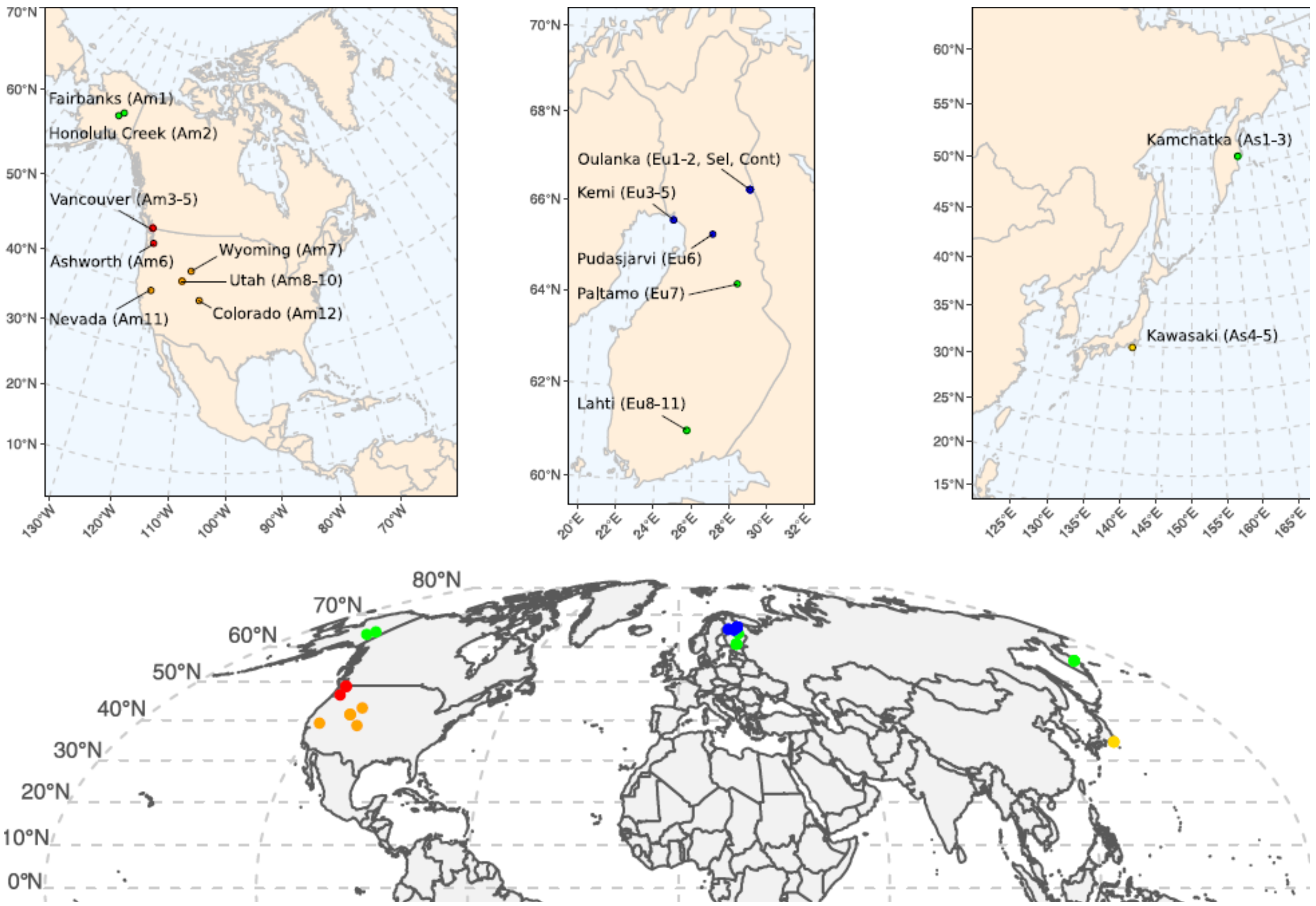

Figure S1. Geographic origin of *D. montana* strains (note different scales).

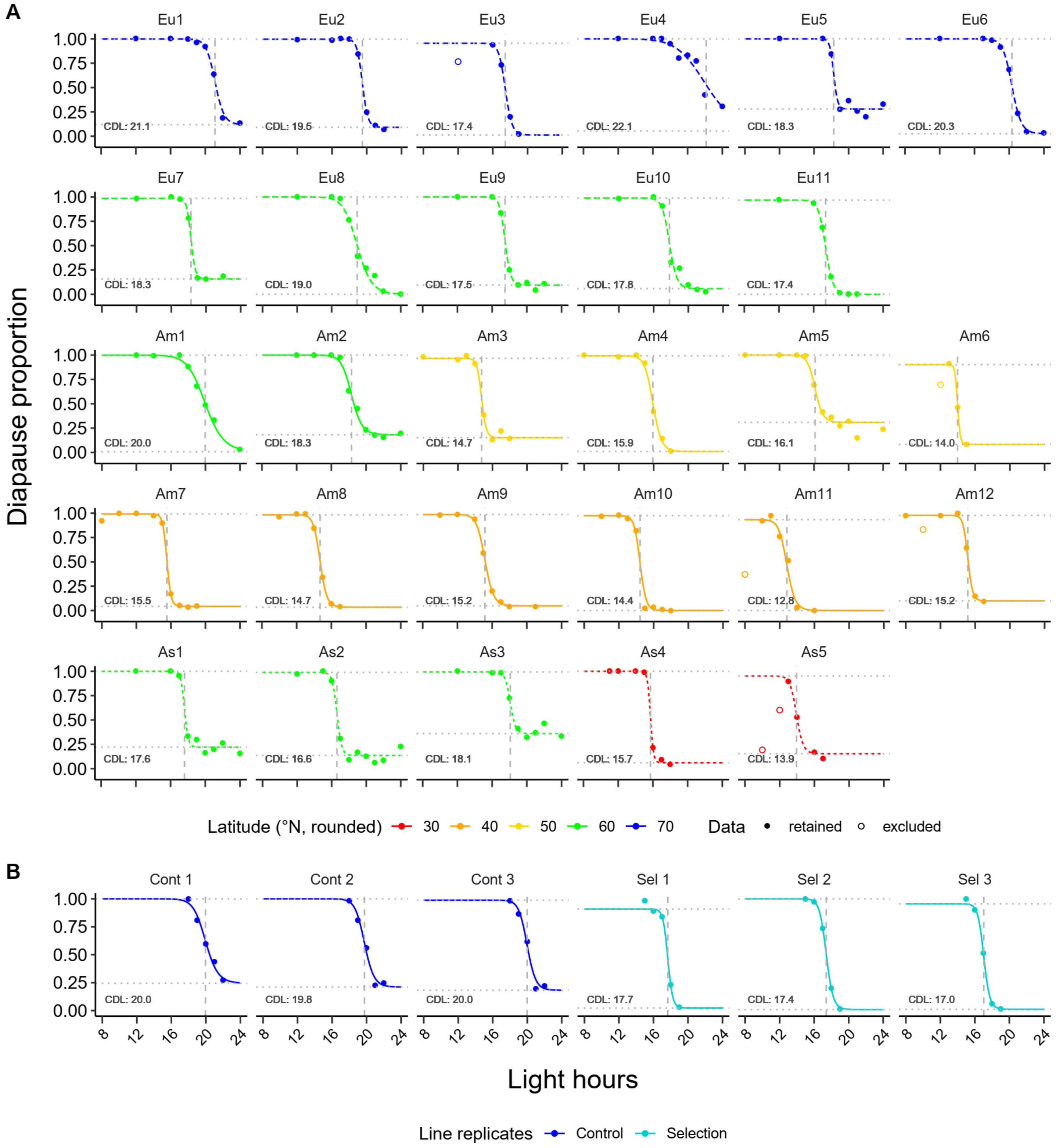

Figure S2. Photoperiodic response curves (PPRCs) used for the estimation of CDLs at 16°C for the fly strains from different latitudes (A) and for the control and selection line replicates from the selection experiment (B). Only the data marked with filled circles were used in CDL estimation (see Methods).

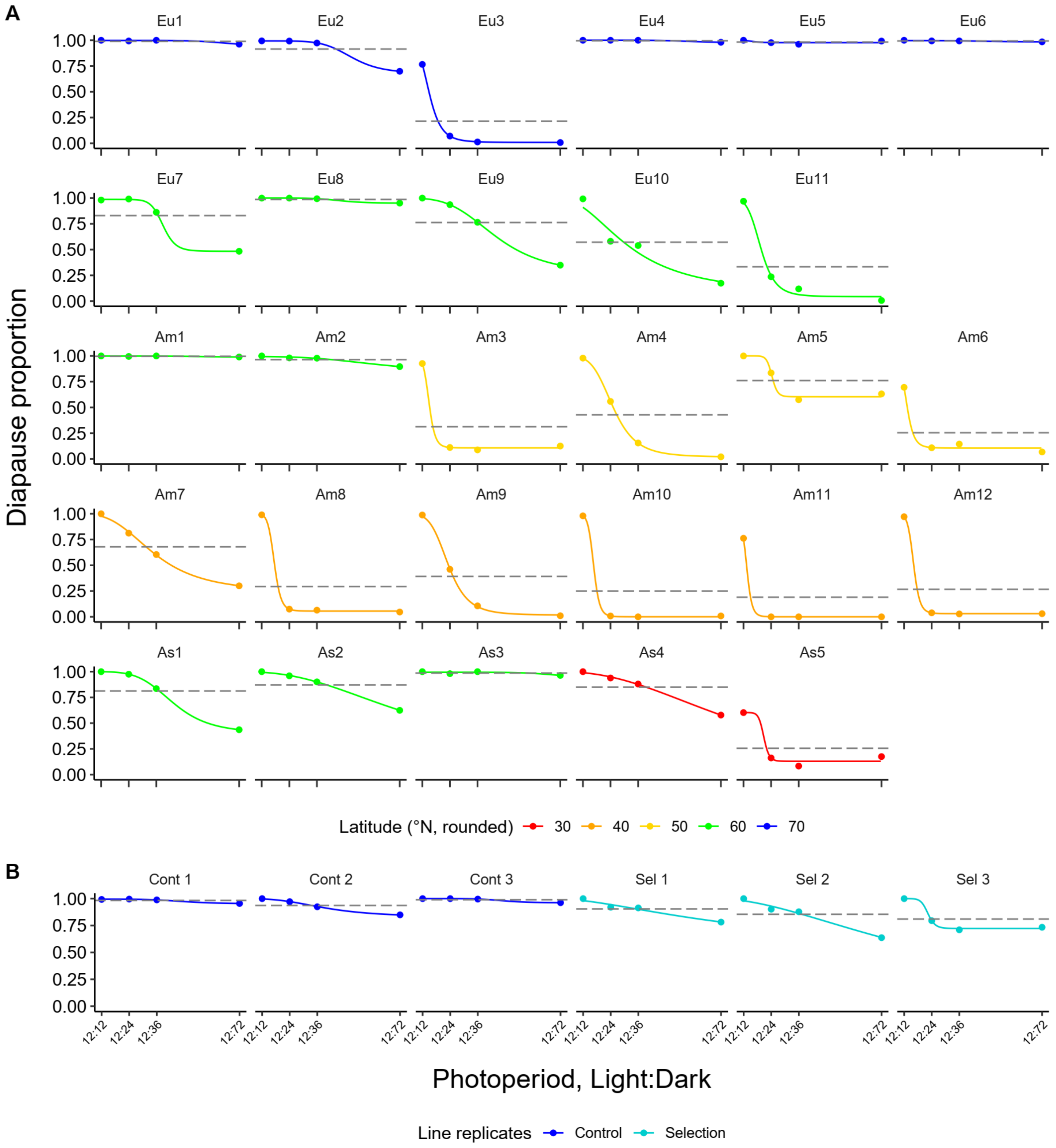

Figure S3. Estimation of the female diapause percentages at 16ºC in NH experiments in ≥ 12 h photoperiods for the fly strains from different latitudes (A) and for the control and selection line replicates from the selection experiment (B).

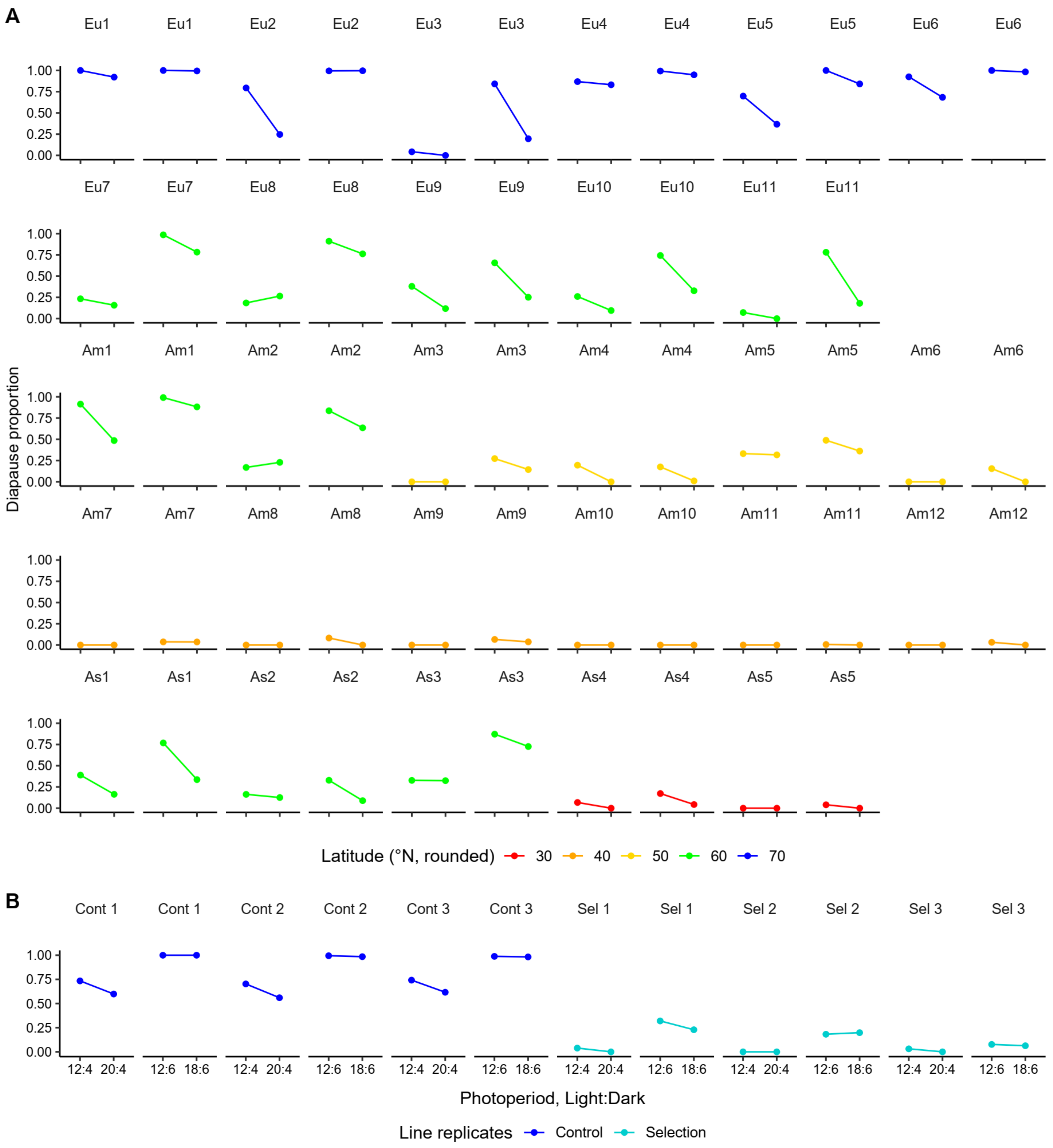

Figure S4. Female diapause proportions at 16ºC in extra short (4 and 6 h) night lengths in NH experiment vs. the same night lengths in 24 h cycles (LD12:4 vs. 20:4) and 6 h (LD 12:6 vs. LD 18:6) for the fly strains from different latitudes (A) and for the control and selection line replicates from the selection experiment (B).

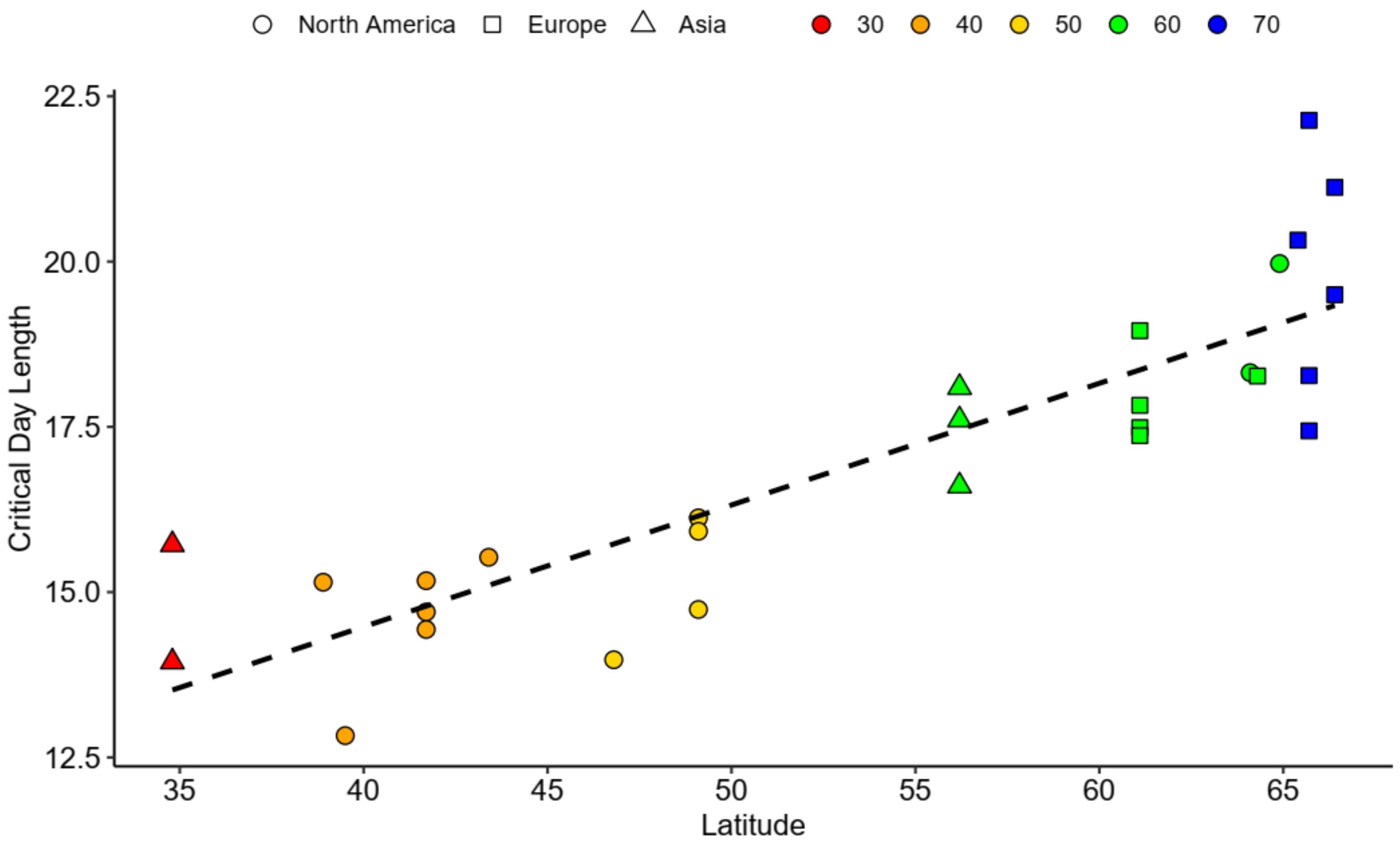

Figure S5. Correlation between the strains' Critical Day Length (CDL) and latitude.

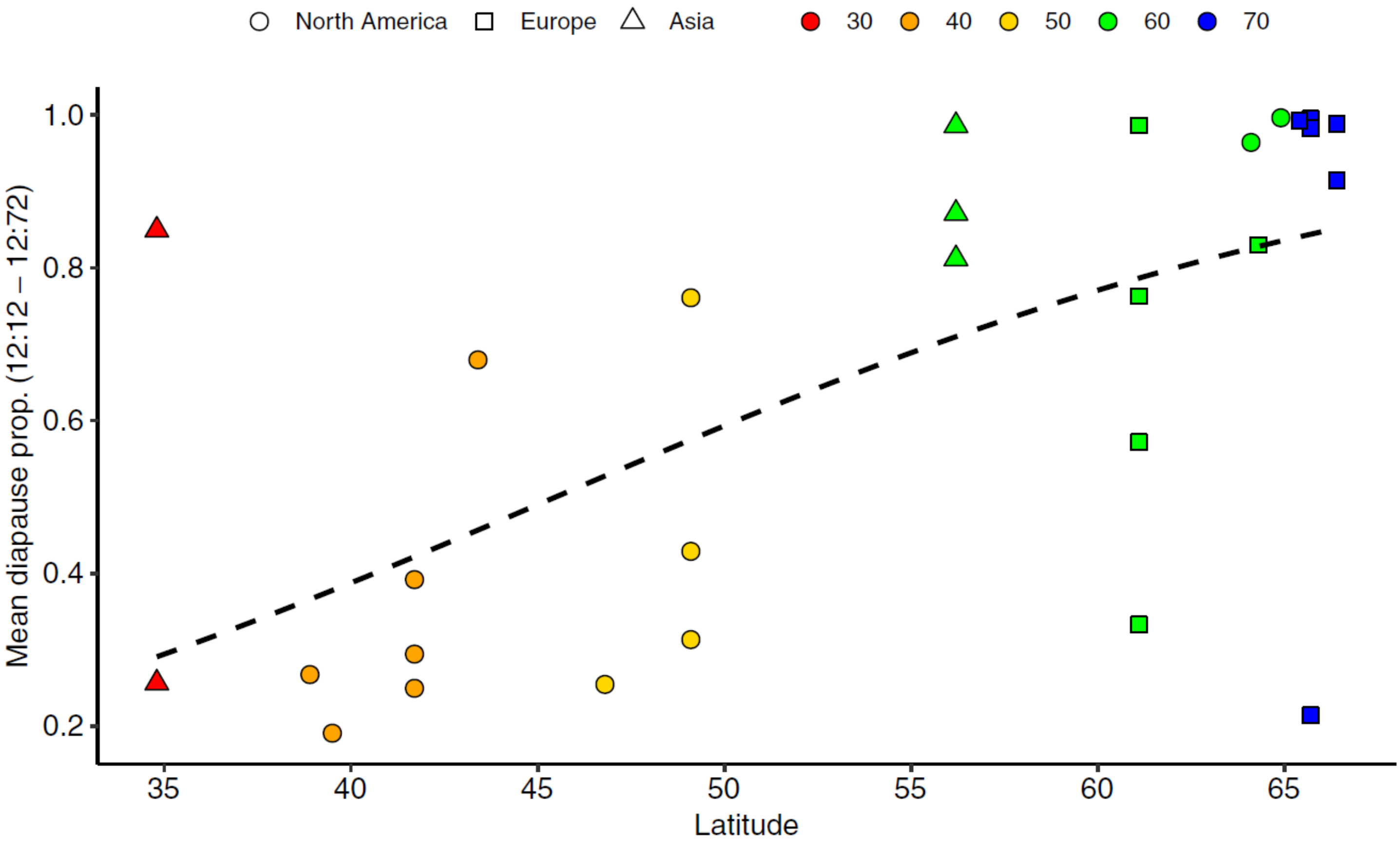

Figure S6. Correlation between the strains' mean diapause proportion in ≥ 24 h photoperiods and latitude.
